# Delayed Presentation of Cerebral Air Embolism from a Left Atrial-Esophageal Fistula: A Case Report and Review of the Literature

**DOI:** 10.7759/cureus.1850

**Published:** 2017-11-15

**Authors:** Catherine Peterson, Clay Elswick, Vicki Diaz, R. Shane Tubbs, Marc Moisi

**Affiliations:** 1 Neurosurgery, Wayne State University School of Medicine; 2 Neurosurgery, Seattle Science Foundation

**Keywords:** altered mental status, atrial esophageal fistula, cerebral air embolism, hyperbaric oxygen therapy, pneumocephalus

## Abstract

Air embolism developing from an atrial-esophageal fistula that was created as a complication from an atrial ablation procedure is a rare, yet usually fatal diagnosis. Neurologic manifestations such as meningitis, altered mental status, seizures, strokes, transient ischemic attacks (TIAs), psychiatric changes, and coma can ensue. Imaging of the brain might reveal infarcts, cerebral edema, as well as signs of pneumocephalus. This case describes a 42-year-old male with recent cardiac ablation procedure at an outside hospital for refractory atrial fibrillation (A-fib) who presented with altered mental status, dyspnea and diaphoresis. His initial head computed tomography (CT) scan revealed pneumocephalus. He was started on a heparin drip for a non-ST elevation myocardial infarction (NSTEMI), but developed severe coagulopathy. The patient’s mental status quickly deteriorated. Given recent cardiac ablation procedure, the cause of his air embolism was thought to be from a created left atrial-esophageal fistula. Despite medical management, he was too unstable to undergo any surgical intervention for his atrial-esophageal fistula or to transfer to a hyperbaric oxygen therapy center, and expired on the second day following his hospital admission. To our knowledge, few reports have been published in the literature describing delayed cerebral air embolism from an atrial-esophageal fistula. Prompt diagnosis, hyperbaric oxygen therapy, and surgical intervention are essential to avoid mortality in these patients. This article aims to increase awareness of such a rare, but significant complication.

## Introduction

Atrial-esophageal fistula is a rare, yet fatal complication of left atrial ablation therapy performed for refractory atrial fibrillation (A-fib). According to a systematic review by Yousuf, et al., the mortality rates for atrial-esophageal fistula range from 33% with surgical intervention to 96% with medical management alone and even 100% with stent placement [1]. It is well known that air embolism can be caused by trauma, barotrauma, or various surgical procedures including cardiac, vascular, and neurosurgery. Atrial-esophageal fistula from recent left atrial ablation therapy can be a source of air emboli into the systemic circulation. Treatment is usually with supplemental oxygen, blocking the communication between the vascular system and atmosphere, and prompt hyperbaric oxygen therapy [2]. A high level of diagnostic suspicion and early surgical intervention are the keys to survivability of patients diagnosed with an atrial-esophageal fistula.

Herein, we report the case of a 42-year-old male presenting with neurologic symptoms due to cerebral air embolism from an atrial-esophageal fistula, a complication of his recent atrial ablation therapy. Physicians should be aware of atrial-esophageal fistula as a source of cerebral air embolism in patients who have recently undergone atrial ablation therapy.

## Case presentation

History

A 42-year-old male was brought to the emergency department (ED) in the evening via ambulance with initial complaints of dyspnea and diaphoresis. Prior to arrival to the ED, the patient had a syncopal episode in the ambulance. The patient had a significant cardiac history of hypertension, congestive heart failure with last ejection fraction of 10%, coronary artery disease, and refractory A-fib with rapid ventricular response. His CHA2DS2-VASc score was four. One month prior he underwent a cardiac ablation procedure at an outside hospital for his refractory A-fib. Once he regained consciousness, he denied any recent trauma, chest pain, headache, or focal neurologic deficits. Of note, he was evaluated at an outside hospital a day prior for similar symptoms with intermittent chest pain, but the workup there was not significant and he was released home.

Physical examination

Upon arrival to the ED, the patient was initially unresponsive, Glasgow coma scale (GCS) of 3, with right gaze deviation. Interestingly, within a few minutes, he regained consciousness and was alert and oriented. He was at 100% oxygenation on room air, but was noted to be tachycardic with heart rate in the 120s. The rest of his vital signs were stable. He was alert and oriented and his pupils were symmetric and reactive. Remaining cranial nerves were intact. Generalized weakness in his extremities was noted. Sensation to light touch and rectal tone were intact.

Hospital course

**Figure 1:**
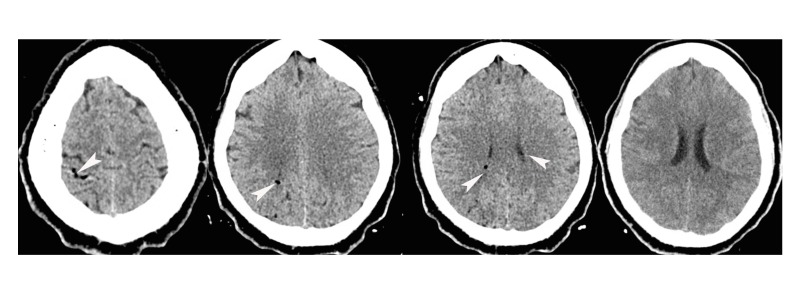
Axial non-contrast computed tomography (CT) of the head. Head CT with arrows point to intravascular pockets of air on several cuts.

In the ED, the patient was placed on a cardiac monitor, oxygen, and intravenous (IV) access was established without difficulty. Electrocardiogram showed sinus tachycardia and initial chest radiograph did not show any significant abnormalities. However, in the ED, the patient became confused again and experienced acute bilateral vision loss with only intact light perception. An emergent head CT was performed which revealed small collections of intravascular air throughout, but was negative for any intracranial hemorrhage, midline shift, mass effect or ventriculomegaly (Figure 1). CT of the thorax/abdomen/pelvis was also obtained and was negative for any arterial injury, dissection, pulmonary embolism or pneumothorax. Decision was made to admit the patient to medical intensive care unit (MICU) due to concerns for an acute air embolism, considering his recent cardiac ablation procedure. Additionally, imaging findings such as pneumomediastinum, pneumopericardium, and/or contrast extravasation from the left atrium into the esophagus are not required to make a probable diagnosis of left atrial-esophageal fistula. The patient’s troponins were also significantly elevated and he was diagnosed with a non-ST elevation myocardial infarction (NSTEMI). He was started on aspirin, Plavix, heparin drip, and the cardiology team was consulted for a possible cardiac catheterization the next morning. He was also kept on a non-rebreather mask and plans were made to transfer him to a hyperbaric oxygen therapy center.

**Figure 2:**
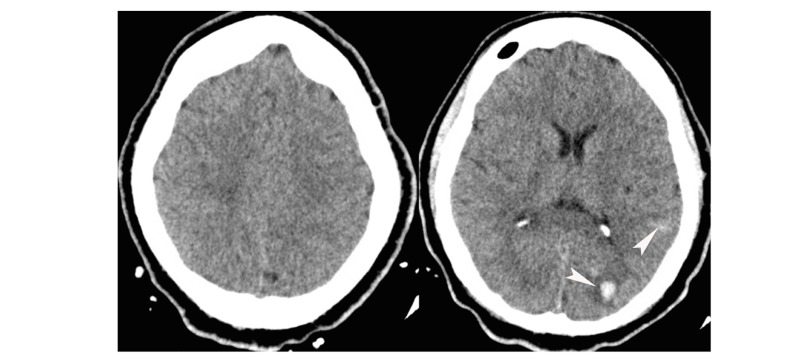
Axial non-contrast computed tomography (CT) of the head. Repeat non-contrast head CT with developing small left parieto-occipital contusions. Arrows point to developing contusions.

After starting heparin drip, the patient then developed two episodes of hematemesis and hematuria and the decision was made to discontinue his heparin drip, aspirin, and Plavix. The next morning, he was tachycardic, febrile and lethargic. On examination, he was oriented only to his name, pupils were equal but sluggishly reactive, and he was able to follow commands. A head CT was repeated which now revealed small developing left parieto-occipital and right frontal contusions without mass effect or midline shift and with slight improvement in pneumocephalus (Figure 2). Neurosurgery recommended beginning levetiracetam, repeating a head CT in six hours, avoiding blood thinners, correcting the patient’s coagulopathy, and prompt transfer to a hyperbaric oxygen therapy center. The patient’s condition unfortunately rapidly deteriorated while in the MICU. His mental status declined and he had to be intubated for airway protection. He became hypotensive and norepinephrine and vasopressin drips were started. Cardiothoracic surgery declined to intervene, as the patient was now too unstable for any surgical intervention. The planned transfer to hyperbaric oxygen therapy center had to be deferred due to instability. The patient had gross bleeding from the rectum as well as from multiple IV access sites, his laboratory results confirmed disseminated intravascular coagulation (DIC), and massive transfusion protocol was initiated. The patient exhibited a GCS of 3T with intact brainstem reflexes. He then suffered a cardiac arrest and expired on the second day from his admission despite medical management. The patient’s family, in this case, declined an autopsy.

## Discussion

Although there are multiple causes of air embolism, it is most often iatrogenic and occurs in a hospital setting. At present, procedures such as endoscopy, angiography, tissue biopsy, thoracocentesis, hemodialysis and venous access contribute to most air embolism cases. A venous gas embolism is created by a pressure gradient that allows air to enter the blood stream, which obstructs the pulmonary vasculature. If an anatomic cardiac defect or large volumes of gas are present, air bubbles can travel through the pulmonary vasculature into the systemic circulation, which then can potentially compromise the blood supply to various organ systems causing ischemia and infarcts. On the other hand, arterial gas embolism forms when air directly enters the arterial circulation. Clinical signs and symptoms depend on the location of the air embolism. A study by McCarthy, et al. found the pulmonary artery to be the most common location for air embolism and cerebral arteries as the second most common location. The same study also revealed that neurologic symptoms and cardiac arrest are predominant manifestations from an air embolism [2].

This case report describes how an atrial-esophageal fistula came to be the source for delayed cerebral air embolism in a patient who underwent left atrial ablation therapy four weeks prior to A-fib. Catheter ablation therapy can cause esophageal complications in up to 47% of patients and atrial-esophageal fistula is the most fatal of those [1]. One nationwide survey concluded that the prevalence of atrial-esophageal fistula was only 0.03% after a left atrial ablation procedure. Nonetheless, all of the patients had some type of major neurological manifestation and more than 80% of them had died [3]. But as atrial ablation therapy for refractory A-fib is gaining popularity, the incidence of various complications is becoming more evident. Esophageal complications in general are quite common from these procedures due to the proximity of the esophagus to the posterior wall of the left atrium [4].

Atrial-esophageal fistula can present as early as three days and as late as five weeks post procedure with symptoms that include but are not limited to fever, chest pain, dysphagia, dyspnea, melena, hematemesis, sepsis and various neurologic symptoms [1]. However, one case report by Giesen, et al. published in 2016 found manifestations from an atrial-esophageal fistula as late as eight weeks following the ablative therapy [5]. Neurologic sequelae often predominate due to cardiac, septic or air emboli. These may include meningitis, altered mental status, seizures, strokes, transient ischemic attacks (TIAs), psychiatric changes and coma. Imaging of the brain might reveal infarcts, cerebral edema, as well as signs of pneumocephalus [4]. Cerebral air emboli and cardiopulmonary compromise are the main etiologies of these neurologic symptoms [2]. Oxygen-free radicals promote edema and inflammation, leading to worsening neurological deficits [6]. Moreover, atrial-esophageal fistula not only introduces air, but also bacteria and food into systemic circulation, resulting in embolization of the brain and multifocal infarcts [7].

If there is high suspicion for atrial-esophageal fistula, an urgent thoracic contrast-enhanced CT or MRI is required and may reveal pneumomediastinum, pneumopericardium, and/or contrast extravasation from the left atrium into the esophagus. However, these findings are not required to make a probable diagnosis of left atrial-esophageal fistula following a recent cardiac intervention as seen in this case report. Of note, it is important to avoid any interventional diagnostic procedures such as transesophageal echocardiogram or esophagoscopy due to potential of introducing air embolism [8]. Sonmez, et al. described a 58-year-old female who presented 22 days after her procedure with fever, chills and right arm numbness. Transesophageal echocardiogram was performed because the transthoracic echocardiogram suggested the presence of an atrial thrombus. The patient’s neurologic status quickly deteriorated after her transesophageal echocardiogram requiring cardiopulmonary bypass and repair of her atrial-esophageal fistula. She died 20 days later due to multi-organ failure [9]. This further summarizes the importance of avoiding such interventional diagnostic tests in patients with clinical suspicion of an atrial-esophageal fistula.

Many of these patients present with fever or signs of sepsis and although antimicrobial therapy is commonly initiated, it should not be the primary therapy. Several cases have shown that septic or air embolization will only continue unless prompt surgical intervention is undertaken [10]. It is crucial to promptly initiate hyperbaric oxygen therapy in patients presenting with cerebral air emboli and neurological deficits. Administering high-flow supplemental oxygen is also important as it increases the rate of absorption of the embolized air bubbles. Additionally, after supportive therapy and stabilization of the patient, surgical repair of the atrial-esophageal fistula should be undertaken [6]. Even in patients whose atrial-esophageal fistula was repaired surgically, few survived and many had lasting neurologic deficits. Due to fever and neurologic manifestations being common, it is important to realize that many of these patients often present to infectious disease or stroke units, highlighting the importance of high clinical suspicion and prompt diagnosis by neurologists and neurosurgeons [10].

## Conclusions

Atrial-esophageal fistula is an extremely rare but one of the most fatal complications of left atrial ablation therapy performed for refractory A-fib. It can potentially introduce air into the systemic circulation, leading to acute cardiopulmonary collapse and various neurologic sequelae. This case report highlights the importance of considering delayed presentation of an atrial-esophageal fistula as a source of cerebral air embolism in patients who have recently undergone atrial ablation therapy and now presenting with signs and symptoms of air embolism. If prompt diagnosis, acute hyperbaric oxygen therapy, and surgical repair are not instituted, the mortality of such complication is expected to be 100%.
